# Variation in dry grassland communities along a heavy metals gradient

**DOI:** 10.1007/s10646-015-1569-7

**Published:** 2015-10-22

**Authors:** Marcin W. Woch, Paweł Kapusta, Anna M. Stefanowicz

**Affiliations:** Institute of Biology, Pedagogical University of Kraków, Podchorążych 2, 31-054 Kraków, Poland; W. Szafer Institute of Botany, Polish Academy of Sciences, Lubicz 46, 31-512 Kraków, Poland

**Keywords:** Historical mining, Heavy metal, Dry grassland, Species richness, Species composition

## Abstract

The aim of this study was to investigate the variation in plant communities growing on metal-enriched sites created by historical Zn–Pb mining. The study sites were 65 small heaps of waste rock covered by grassland vegetation and scattered mostly over agricultural land of southern Poland. The sites were described in terms of plant coverage, species richness and composition, and the composition of plant traits. They were classified using phytosociological methods and detrended correspondence analysis. Identified plant communities were compared for vegetation parameters and habitat properties (soil characteristics, distance from the forest) by analysis of variance. The variation in plant community parameters was explained by multiple regression, in which the predictors were properties of the habitat selected on the basis of factor analysis. Grasslands that developed at low and high concentrations of heavy metals in soil were similar to some extent: they were composed on average of 17–20 species (per 4 m^2^), and their total coverage exceeded 90 %. The species composition changed substantially with increasing contamination with heavy metals; metal-sensitive species withdrew, while the metal-tolerant became more abundant. Other important predictors of community structure were: proximity to the forest (responsible for the encroachment of competitive forest species and ruderals), and the thickness of the surface soil (shallow soil favored the formation of the heavy metal grassland). The heavy metal grassland was closely related to the dry calcareous grasslands. The former was an earlier succession stage of the latter at low contamination with heavy metals.

## Introduction

Heavy metals present in the soil at elevated concentrations can be the overriding factor of the plant species distribution. Numerous studies on the vegetation of metalliferous sites showed that plant species richness and composition changed dramatically under the influence of soil contamination with arsenic, cadmium, chromium, copper, lead, mercury, nickel or zinc (e.g. Simon 1978; Clark and Clark 1981; Lejeune et al. 1996; Brown 2001; Proctor 2003; Strandberg et al. 2006; Becker and Brändel 2007; Válega et al. 2008; Myking et al. 2009; Lucassen et al. 2010; Mapaure et al. 2011; Pandey et al. 2015). Since heavy metals are persistent contaminants, they affect the structure of plant communities not only on a short time scale, through the elimination of stress-sensitive species, but also in the long term, by exerting a strong selective pressure on local populations leading to the emergence of new metal-tolerant ecotypes/varieties (Prasad and Hagemeyer 1999; Ernst 2006; Baker et al. 2010).

The most conspicuous effect of microevolution induced by heavy metals is endemic metallophytes. They are obligate metallophytes, i.e. they have developed various adaptations to cope with metal toxicity and are restricted to metal-enriched habitats (Kruckeberg and Kruckeberg 1989; Prasad and Hagemeyer 1999). Currently, a high rate of population decline of these species is recorded, which provokes actions towards their conservation (Whiting et al. 2004; Baker et al. 2010; Baumbach 2012). Other metallophytes are more cosmopolitan (facultative metallophytes). They are derived from common species and, owing to their great genetic and phenotypic plasticity, can colonize both metalliferous and non-metalliferous soils (Ernst 2006).

The residual nature, extreme fragmentation and dispersion, and a great geochemical diversity of metal-enriched habitats as well as floristic peculiarity of some (the occurrence of very rare endemics) make it difficult to perform large-scale studies that could improve our understanding of the European heavy metal grassland. Studies concerning the relationship between the concentration of heavy metals in soil and plant species distribution are usually based on a fairly dense sampling of single and relatively small areas (Simon 1978; Brown 1994; Strandberg et al. 2006; Becker and Brändel 2007; Grodzińska and Szarek-Łukaszewska 2009). The effect of heavy metals on vegetation can be difficult to assess in these types of works, because it is often partly related to other soil characteristics, such as pH, Ca concentration, or thickness of organic layer (Simon 1978; Brown 1994; Becker and Brändel 2007). Moreover, strong connections (short distances) between the surveyed vegetation patches may significantly bias data by spatial autocorrelation—the structure of a plant community recorded in a given patch may result not only from the local environmental conditions but also community processes occurring in neighboring patches, such as plant dispersal or contagion (Dormann et al. 2007). This complicates statistical analysis and interpretation of the results.

To understand what happens in grassland communities with increasing concentrations of heavy metals in soil, one could establish many sites in a wide gradient of metal contamination (other habitat parameters should not vary to avoid interactions), and the sites should be separated by appropriate distances (to meet the assumption of independence). Such ideal conditions rarely occur in the field. However, we managed to find them in the industrial part of western Małopolska (S. Poland). This region is known for its rich Zn–Pb ore deposits, which have been mined since the Middle Ages (Stefanowicz et al. 2014). In our recent study (Stefanowicz et al. 2014), we surveyed nearly 750 km^2^ of the region searching for the remnants of historical Zn–Pb mining. We focused on small heaps of calcareous gangue (waste material) left at the old open pits and shafts. These heaps are termed locally *warpie*. Many of these sites were afforested. Fortunately, some survived relatively intact on agricultural land, and now they are covered by dry grassland vegetation. They all are metalliferous sites, because the concentrations of heavy metals in the soil are elevated compared to environmental standards. These concentrations vary considerably. According to our measurements, the levels of particular heavy metals ranged as follows: 5–522 mg Cd kg^−1^, 0.1–23 g Pb kg^−1^, 6–51 mg Tl kg^−1^, and 0.4–70 g Zn kg^−1^ (Stefanowicz et al. 2014). Large fluctuations in metal contamination between sites likely result from the different ore concentration or function of a given shaft (exploitation, exploration, ventilation). Interestingly, metal pollution is the only steep environmental gradient present there—other soil parameters, such as texture, pH, organic matter or nutrient concentration do not vary significantly, which is probably caused by the same history of origin and bedrock material. Distances between neighboring sites can be large (several km) or small (a few hundred meters). However, even in the latter case, they form isolated islands in the landscape in terms of both geology and vegetation. Thus, they can be considered as independent samples.

The present study used 65 of these sites to answer several questions concerning the formation of heavy metal grassland, namely: (1) in what range of the soil concentrations of Cd, Pb and Zn does this grassland develop; (2) what are the most important differences between the metal-tolerant and metal-sensitive grasslands in terms of species richness and composition; and (3) to what extent does the grassland vegetation respond to variation in other parameters of the habitat?

## Materials and methods

### Study area

**Fig. 1:**
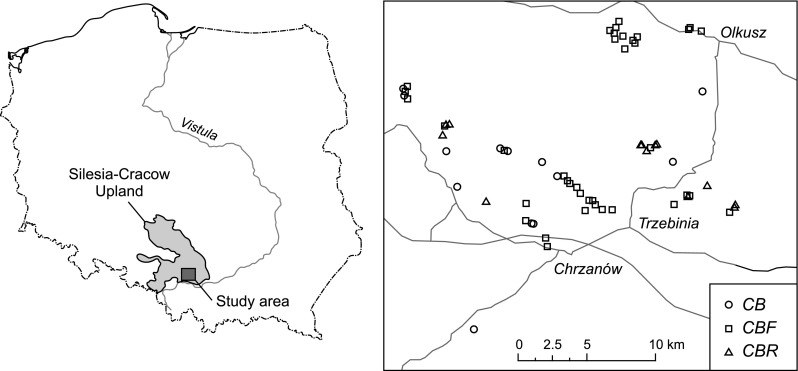
Location of study area in Poland and 65 study sites. Sites were marked with * different symbols* according to the type of grassland: the typical variant of *Carlino acaulis*-*Brometum erecti* (*CB*), the *Festuca ovina* variant (*CBF*) and the *Rubus caesius* variant (*CBR*). Major cities and main roads are also indicated

Study sites, 65 metal-rich mining waste heaps covered by grassland vegetation (Stefanowicz et al. 2014), were located in western Małopolska (S Poland), between the towns of Olkusz, Krzeszowice, Libiąż and Jaworzno (Fig. 1). In this area, the main geological formations are carbonate rocks of the Triassic and Jurassic period, partially covered by Pleistocene fluvioglacial sands (Cabała et al. 2008). The climate is transitional between temperate oceanic in the west and temperate continental in the east. The average annual air temperature fluctuates between 7.1 and 8.1 °C, and the range of the average annual rainfall is 700–873 mm. The growing season spans between 205 and 215 days (Lorenc 2005). A large part of the area (45 %) is covered by forest. Artificially introduced pine monocultures dominate; impoverished beech forest as well as riparian deciduous forest also occur but are less common. Other types of vegetation include segetal and ruderal communities, meadows, wetlands, warm border grasslands and psammophilous grasslands (Tokarska-Guzik 1999; Cohn et al. 2001; Suder and Cabała 2004; Woch 2011; Woch et al. 2013).

### Fieldwork

Vegetation was sampled during the 2012 and 2013 growing seasons, from April to October, on 4-m^2^ circular plots. Plots were established on the southern, south-eastern or south-western slopes of heaps (one study plot per heap) in homogenous patches of grassland. The cover of vascular plant species for each plot was estimated on a five-degree Braun-Blanquet scale (1964). This measurement was based on at least two visits per plot in each year to ensure that all species had been recorded. Although all heaps were situated in the open area, some of them were near the forest and the influence of woody vegetation (e.g. shadowing) could not be excluded. Therefore, the distance to the nearest forest patch (at least 0.1 ha in size) was measured for each plot directly in the field or by using aerial photographs. In July, three topsoil samples were taken from each heap to a depth of 15 cm (or less, if the surface soil horizon was shallow) and bulked into one composite sample. The three sampling spots were at the edge of the study plots so as not to disturb plot vegetation. For these sampling spots, the thickness of the organic matter horizon (O horizon) and the surface soil horizon (A or transitional AC horizon) was measured in the field, according to Mocek et al. (2004), and averaged per plot. All heaps were positioned using GPS.

### Vegetation data handling

Plant nomenclature followed Mirek et al. (2002). Plant communities were identified according to Chytrý (2007). For each plot, species richness (total number of plant species) and the number of species representing different types of seed dispersal (Podbielkowski 1995), Grime’s CSR strategies (Grime 2001), functional groups (Rutkowski 2008), life forms (Raunkiaer 1934), and vegetation classes (Chytrý 2007, 2009, 2013) were calculated.

### Soil analysis

Prior to the analysis, soil samples were sieved (2 mm mesh) and dried at 105 °C. The content of sand, silt and clay was determined by a combination of sieving and sedimentation, and the soil pH was tested with a pH-meter after extraction with water in a 1:5 (w:v) ratio. Organic carbon was assessed using the dry combustion technique with a Leco RC-612. Total sulfur was measured with a Leco SC-144 DR. Total nitrogen was determined using the Kjeldahl method. The soil was digested in H_2_SO_4_ with Kjeltabs (K_2_SO_4_ + CuSO_4_·5H_2_O; Foss Tecator Digestor Auto) followed by distillation in a Foss Tecator Kjeltec 2300 Analyser Unit. To determine the concentration of pseudo-total (hereafter referred to as “total”) and EDTA metals, two soil extraction methods were applied: hot concentrated HClO_4_ (Foss Tecator Digestor Auto; Ca, Cd, K, Mg, Pb, Tl, Zn) and 0.05 M EDTA (Cd, Pb, Tl, Zn). The metal concentrations in soil extracts were analyzed by flame atomic absorption spectrometry (Varian 220 FS). The total phosphorus was measured using the molybdenum blue method with a colorimeter (Hach-Lange DR 3800) after digestion in hot HClO_4_. Available (Olsen) phosphorus was measured with an ion chromatograph (Dionex ICS-1100) following the extraction of soil with 0.5 M NaHCO_3_, pH 8.5.

### Data analysis

Prior to statistical analyses, the variables (properties of habitat and vegetation parameters) were transformed with a logarithmic or exponential function, according to the formulas proposed by Økland et al. (2001), and expressed on a 0–1 scale in order to achieve homogeneity of variances (Økland 2007). Detrended correspondence analysis (DCA) was applied for the plant data to identify important ecological gradients determining plant species distribution and to verify the classification of the plots performed with phytosociological methods. One-way ANOVA (or Kruskal–Wallis test, in case of violation of the assumptions of normality and homogeneity of variance) was used to compare habitat properties and vegetation parameters between plant community groups identified by the phytosociological analysis. Factor analysis with a varimax rotation was performed to reveal interrelationships between soil variables and obtain a small number of uncorrelated factors representing the main sources of variation in the soil dataset. One variable (O horizon thickness) was excluded from this analysis due to its distribution, which could not be normalized. The number of factors was established on the basis of Kaiser’s criterion (eigenvalue >1). Factors were used together with the distance from the forest (DFF) in multiple regression (with forward stepwise selection procedure) as independent representatives of habitat conditions to explain plant species richness and composition (scores for axes 1 and 2 in DCA). Analyses were carried out using STATISTICA 9 (StatSoft Inc. Tulsa, OK, USA) and CANOCO 4.5 software (ter Braak and Šmilauer 2002).

## Results

### Grassland vegetation of old mining heaps

Plant communities included in this study were dry calcareous grasslands of the *Carlino acaulis*-*Brometum erecti* association (the *Bromion erecti* alliance, the *Festuco*-*Brometea* class). They occurred in three variants: (1) closed grassland dominated by *Brachypodium pinnatum* (typical variant, *CB*), (2) loose heavy metal grassland with *Festuca ovina* as the main component (the *Festuca ovina* variant, *CBF*), and (3) grassland with woody and ruderal plants (the *Rubus caesius* variant, *CBR*).

Vegetation typical of *Carlino acaulis*-*Brometum erecti* (*CB*) was found on 13 heaps. It was strongly dominated by *Brachypodium pinnatum*. Other frequent components of the *CB* community were *Euphorbia cyparissias*, *Lotus corniculatus*, *Achillea collina*, *Galium album*, *Medicago falcata* and *Peucedanum oreoselinum*. The *CB* plots were almost entirely covered with vegetation and contained an average of nearly 20 species, most of which belonged to the *Festuco*-*Brometea* class (Table 1). This community was distinguished by a higher than elsewhere number of zoochorous plants and stress-tolerant competitive plants.

**Table 1:** Plant community characteristics averaged (mean ± standard deviation) for the three types of vegetation (expressed as number of species with the exception of cover)

Variable	*CB* (N = 13)	*CBF* (N = 38)	*CBR* (N = 14)
Cover (%)*	96.5 ± 5.9^ab^	90.5 ± 11.5^a^	98.2 ± 5.4^b^
Species richness	19.6 ± 5.6	20.3 ± 6.0	16.9 ± 4.8
Forb	12.3 ± 4.0^ab^	13.6 ± 4.2^a^	9.9 ± 3.5^b^
Grass	4.1 ± 1.4	4.0 ± 1.9	3.9 ± 1.7
Legume	2.7 ± 0.9	2.1 ± 1.2	1.9 ± 0.9
Woody plant*	0.5 ± 0.5^ab^	0.5 ± 0.8^a^	1.3 ± 1.1^b^
C (competitor)	3.9 ± 2.0^a^	4.3 ± 1.9^a^	7.5 ± 2.3^b^
CR (competitive ruderal)*	0.5 ± 0.7	0.8 ± 0.9	0.6 ± 0.8
CSR (mixed strategy)	9.7 ± 4.4^a^	10.7 ± 3.8^a^	5.6 ± 3.4^b^
R (ruderal)*	0.2 ± 0.6	0.1 ± 0.2	0.1 ± 0.3
S (stress tolerator)*	0.3 ± 0.5^ab^	0.8 ± 0.6^a^	0.1 ± 0.5^b^
SC (stress-tolerant competitor)	4.8 ± 1.3^a^	3.3 ± 1.9^ab^	3.0 ± 1.5^b^
SR (stress-tolerant ruderal)*	0.2 ± 0.4	0.2 ± 0.5	0.0 ± 0.0
Chamaephyte	1.5 ± 1.3^a^	3.1 ± 1.1^b^	1.3 ± 1.3^a^
Geophyte	2.5 ± 1.0	3.0 ± 1.2	3.3 ± 1.7
Hemicryptophyte	16.6 ± 4.4	16.1 ± 5.2	12.6 ± 4.1
Liana*	0.1 ± 0.3	0.0 ± 0.0	0.1 ± 0.3
Phanerophyte*	0.7 ± 0.8	0.7 ± 0.9	1.4 ± 1.2
Terophyte*	0.7 ± 0.9	0.5 ± 0.7	0.5 ± 0.5
Anemochory	14.2 ± 4.3^ab^	15.2 ± 4.3^a^	11.7 ± 2.9^b^
Antropochory*	0.5 ± 0.8	0.6 ± 0.6	0.9 ± 0.9
Autochory	2.6 ± 0.8	2.4 ± 1.5	1.9 ± 1.1
Hydrochory*	0.5 ± 0.5	0.1 ± 0.3	0.2 ± 0.4
Myrmecochory*	3.4 ± 1.6	3.0 ± 1.7	2.5 ± 2.3
Zoochory	3.8 ± 2.1^a^	2.0 ± 1.7^b^	3.2 ± 1.6^ab^
*Artemisietea vulgaris*	0 ± 0^a^	0.2 ± 0.4^a^	1.9 ± 1^b^
*Asplenietea trichomanis*	0 ± 0^a^	0.6 ± 0.5^b^	0.1 ± 0.4^a^
*Calluno*-Ulicetea*	0.2 ± 0.4	0.4 ± 0.7	0.1 ± 0.3
*Carpino*-*Fagetea*	0.4 ± 0.5^ab^	0.1 ± 0.3^a^	0.6 ± 0.5^b^
*Epilobietea angustifolii*	0 ± 0^a^	0.1 ± 0.3^a^	0.8 ± 0.6^b^
*Festuco*-*Brometea*	12.9 ± 3.8^a^	11.4 ± 4.4^a^	7.6 ± 3.4^b^
*Koelerio glaucae*-*Corynephoretea canescentis*	0.5 ± 0.7^a^	1.2 ± 0.7^b^	0.1 ± 0.4^a^
*Molinio*-Arrhenatheretea*	4.5 ± 2.4	5.5 ± 2	4 ± 1.7
*Quercetea robori*-*petraeae*	0 ± 0^a^	0.1 ± 0.3^a^	0.6 ± 0.5^b^

*CB* typical variant of *Carlino acaulis*-*Brometum erecti*, *CBF* the *Festuca ovina* variant, *CBR* the *Rubus caesius* variant. Statistically significant (*P* < 0.05) differences between the groups were detected using one-way ANOVA, followed by Tukey’s test or the Kruskal–Wallis test, followed by Dunn’s test (variables analyzed with a non-parametric test were asterisked). Means labelled with different letters are statistically different

The *CBF* variant was most abundant in this study (N = 38). Its most dominant species was the metal-tolerant grass, *Festuca ovina*. The *CBF* plots were overgrown mainly by xerophilous plants, such as *Euphorbia cyparissias*, *Potentilla arenaria*, *Thymus pulegioides* and *Scabiosa ochroleuca*. They had on average a similar number of species as the *CB* plots, but were more open in terms of vegetation cover (Table 1). The *Festuco*-*Brometea* plants were dominant, but contrary to the *CB* variant, they were accompanied by plants of many other vegetation classes. The *CBF* variant was also characterized by the highest number of anemochorous plants, forbs and chamaephytes as well as sporadic but statistically significant presence of stress-tolerators (Table 1). The members of this community were facultative metallophytes, such as *Asperula cynanchica*, *Cardaminopsis halleri*, *Dianthus carthusianorum*, *Gypsophila fastigiata* and *Silene vulgaris*, which were often absent in other types of grasslands.

The *CBR* variant occupied 14 heaps. This grassland had fully closed vegetation (Table 1). It contained an average of 17 species, among which the most frequent were *Rubus caesius*, *Festuca rubra* and *Galium album*. An important role in this community was played by synanthropic species of wide ecological amplitude from the classes of *Artemisietea vulgaris* and *Epilobietea angustifolii*, e.g. *Calamagrostis epigejos*, *Pteridium aquilinum* or *Solidago canadensis*. Some sites were dominated by one or two of these species. In comparison to the two other grassland types, the *CBR* variant had the lowest number of the *Festuco*-*Brometea* plants, anemochorous plants and forbs, while the number of competitors such as woody plants was the highest (Table 1). Another feature of this community was the presence of forest species from the *Carpino*-*Fagetea* class, including *Melica nutans*.

**Fig. 2:**
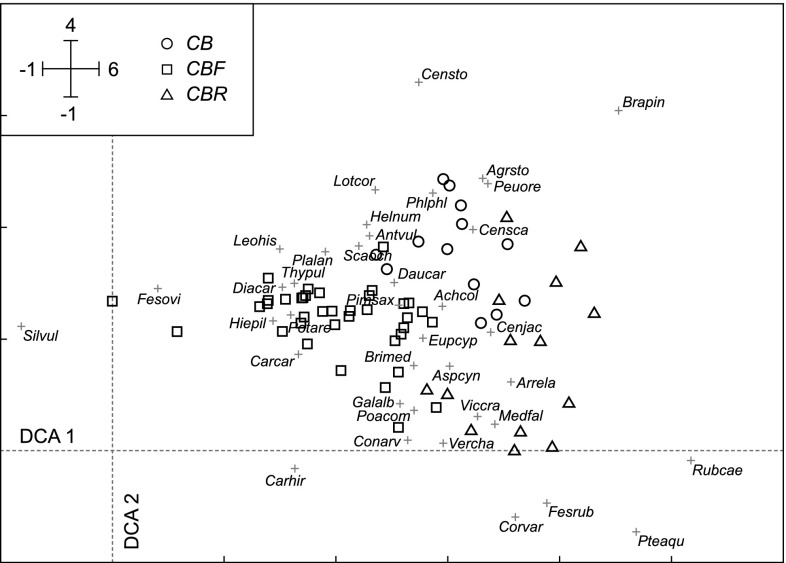
DCA ordination diagram of 65 vegetation samples. *Different symbols* indicate the three types of grassland: the typical variant of *Carlino acaulis*-*Brometum erecti* (*CB*), the *Festuca ovina* variant (*CBF*) and the *Rubus caesius* variant (*CBR*). Species, which occurred infrequently (arbitrarily, <10 % of sites) were not included in this analysis. Diagram shows only important differential species (weight higher than 10 %): *Achillea collina* (*Achcol*), *Agrostis stolonifera* (*Agrsto*), *Anthyllis vulneraria* (*Antvul*), *Arrhenatherum elatius* (*Arrela*), *Asperula cynanchica* (*Aspcyn*), *Brachypodium pinnatum* (*Brapin*), *Briza media* (*Brimed*), *Carex caryophyllea* (*Carcar*), *Carex hirta* (*Carhir*), *Centaurea jacea* (*Cenjac*), *Centaurea scabiosa* (*Censca*), *Centaurea stoebe* (*Censto*), *Convolvulus arvensis* (*Conarv*), *Coronilla varia* (*Corvar*), *Daucus carota* (*Daucar*), *Dianthus carthusianorum* (*Diacar*), *Euphorbia cyparissias* (*Eupcyp*), *Festuca ovina* (*Fesovi*), *Festuca rubra* (*Fesrub*), *Galium album* (*Galalb*), *Helianthemum nummularium* (*Helnum*), *Hieracium pilosella* (*Hiepil*), *Leontodon hispidus* (*Leohis*), *Lotus corniculatus* (*Lotcor*), *Medicago falcata* (*Medfal*), *Peucedanum oreoselinum* (*Peuore*), *Phleum phleoides* (*Phlphl*), *Pimpinella saxifraga* (*Pimsax*), *Plantago lanceolata* (*Plalan*), *Poa compressa* (*Poacom*), *Potentilla arenaria* (*Potare*), *Pteridium aquilinum* (*Pteaqu*), *Rubus caesius* (*Rubcae*), *Scabiosa ochroleuca* (*Scaoch*), *Silene vulgaris* (*Silvul*), *Thymus pulegioides* (*Thypul*), *Veronica chamaedrys* (*Vercha*), *Vicia cracca* (*Viccra*)

The three variants occupied distinctly different but not isolated positions along the ecological gradients defined by DCA axes (Fig. 2). The *CBF* grasslands were shifted to the left in relation to the *CB* and *CBR* grasslands. The latter two were vertically spaced apart, as *CB* took the upper, and *CBR* the lower side of the DCA diagram. The first and second axis explained 13.4 % (eigenvalue = 0.39) and 7.3 % (eigenvalue = 0.22) of the variation in the species composition, respectively.

### Variability of old mining heap habitats

There were significant differences observed between the heaps overgrown by the three grassland communities (Table 2). The *CBF* heaps had large amounts of Cd, Pb and Zn in soil (both total and EDTA-extractable), while the *CB* and *CBR* heaps were only slightly or moderately contaminated with these metals. The latter two differed in the distance from the forest (DFF): *CB* heaps were situated quite far from the forest, while *CBR* heaps almost always bordered on forest. *CBF* heaps were intermediate in this respect. Other considerable differences were found for the total concentration of S (the highest on the *CBF* plots) and available P (the highest on the *CBR* plots).

**Table 2:** Habitat properties averaged (mean ± standard deviation) for the three vegetation types

Variable	*CB* (N = 13)	*CBF* (N = 38)	*CBR* (N = 14)
DFF (m)	175 ± 150^a^	70 ± 94^ab^	28 ± 58^b^
O horizon thickness (cm)*	1.9 ± 2.5	1.6 ± 1.9	2.4 ± 1.9
A/AC horizon thickness (cm)	11.4 ± 4.8	7.2 ± 4.8	12.3 ± 9.0
pH	8.2 ± 0.3	8.1 ± 0.3	7.9 ± 0.4
Sand (%)	71.2 ± 8.6	64.3 ± 10.5	65.4 ± 10.8
Silt (%)	16.5 ± 6.0	21.1 ± 7.9	21.4 ± 9.3
Clay (%)	12.2 ± 6.8	14.6 ± 6.5	13.2 ± 4.1
C_ORGANIC_ (%)	4.39 ± 1.94	5.50 ± 3.20	4.69 ± 2.49
N (%)	0.322 ± 0.135	0.327 ± 0.143	0.316 ± 0.100
S (%)	0.080 ± 0.031^a^	0.198 ± 0.177^b^	0.076 ± 0.032^a^
P (mg kg^−1^)*	641 ± 214	697 ± 104	745 ± 158
P_OLSEN_ (mg kg^−1^)	3.24 ± 2.51^ab^	2.53 ± 1.53^a^	4.79 ± 2.06^b^
K (g kg^−1^)	1.72 ± 1.58	1.90 ± 1.18	1.40 ± 0.72
Ca (g kg^−1^)*	59.8 ± 55.7	67.3 ± 34.0	45.0 ± 32.6
Mg (g kg^−1^)	20.2 ± 19.7	29.2 ± 16.0	23.3 ± 19.0
Cd (mg kg^−1^)*	17 ± 14^a^	127 ± 107^b^	18 ± 9^a^
Pb (g kg^−1^)*	0.58 ± 0.66^a^	4.07 ± 4.60^b^	0.68 ± 0.38^a^
Zn (g kg^−1^)	2.8 ± 2.3^a^	22.3 ± 19.1^b^	3.3 ± 2.1^a^
Tl (mg kg^−1^)	14.1 ± 7.5	20.8 ± 9.1	13.8 ± 5.8
Cd_EDTA_ (mg kg^−1^)*	7.8 ± 6.3^a^	44.4 ± 40.8^b^	6.8 ± 3.0^a^
Pb_EDTA_ (g kg^−1^)*	0.25 ± 0.21^a^	1.31 ± 1.57^b^	0.29 ± 0.27^ab^
Zn_EDTA_ (g kg^−1^)*	0.56 ± 0.97^a^	2.58 ± 2.81^b^	0.32 ± 0.12^a^
Tl_EDTA_ (mg kg^−1^)	0.96 ± 0.73	0.96 ± 0.52	0.74 ± 0.44

*CB* typical variant of *Carlino acaulis*-*Brometum erecti*, *CBF* the *Festuca ovina* variant, *CBR* the *Rubus caesius* variant, *DFF* distance from the forest. Statistically significant (*P* < 0.05) differences between the groups were detected using one-way ANOVA, followed by Tukey’s test or the Kruskal–Wallis test, followed by Dunn’s test (variables analyzed with a non-parametric test were asterisked). Means labelled with different letters are statistically different

**Fig. 3:**
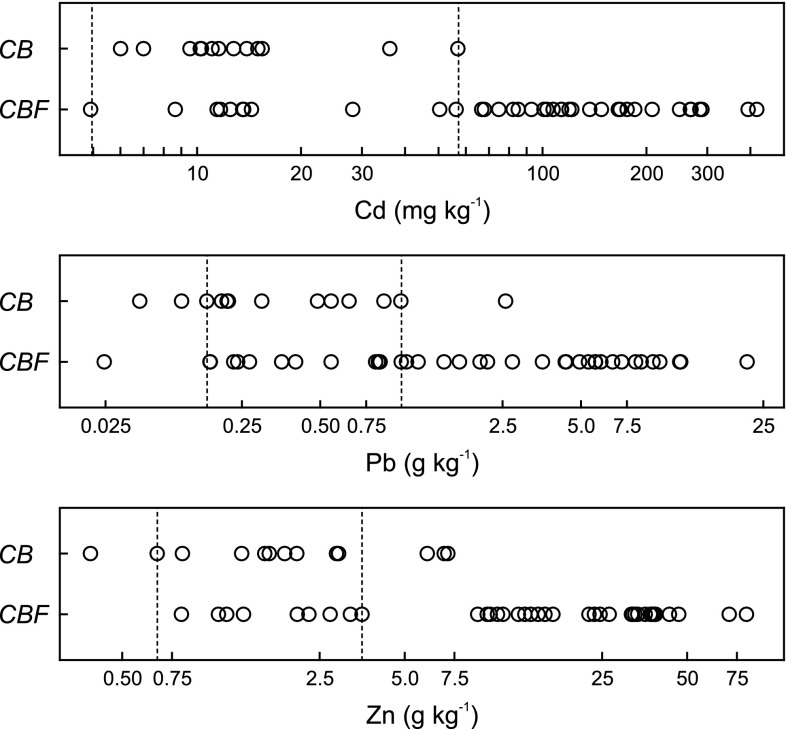
Raw data for the total concentration of Cd, Pb and Zn in soils under typical vegetation of *Carlino acaulis*-*Brometum erecti* (*CB*) and its *Festuca ovina* variant (*CBF*) presented on logarithmic scale. The *dashed lines* indicate the range of metal concentration, within which *CB* and *CBF* sites were compared in terms of natural habitat properties. The ranges were set to obtain homogeneity of variance and insignificant differences in a given metal concentration between the two groups of sites (see the text for explanation)

Although *CBF* soil was on average more contaminated than others, part of the *CBF* sites (ca. one third) fell into the common range of low and moderate levels of Cd, Pb and Zn together with *CB* sites. For example, the concentration of total Cd in *CB* soil varied from 6 to 57 mg kg^−1^. There were 11 *CBF* sites with a similar range of values (Fig. 3). These sites were compared (Student’s *t* test for independent samples) with *CB* sites to identify the factors other than heavy metal contamination that could be responsible for the development of two variants (typical and metal-tolerant) of grassland communities. The results of these comparisons demonstrated that the thickness of A/AC horizon, together with the composition of soil particles, were the only variables explaining the division into the *CB* and *CBF* categories; the *CB* sites had almost two times thicker the A/AC horizon (12 cm), with a slightly higher proportion of sand particles than the *CBF* sites (7 cm).

A factor analysis reduced 21 physicochemical properties of the soil to 6 factors explaining 83 % of the variance in the soil data (Table 3). Factor 1 (F1) represented heavy metal contamination, since it grouped concentrations of both total and EDTA-extractable Cd, Pb and Zn. Factor 2 (F2) comprised total Ca, Tl and Mg, and negatively correlated with these metals, the thickness of A/AC horizon; thus, F2 reflected the amount of weathered calcareous gangue in the sampled soil. Other factors represented organic matter content (F3), soil particle composition (F4 and F5), and pH, which negatively correlated with total P (F6).

**Table 3:** The results of the factor analysis of soil properties (see Table 2 for explanation of variables)

Factor	Variance explained (%)	Variables (with the highest loading values)
F1	28.9	Cd_EDTA_ (0.948), Cd (0.934), Zn (0.921), Zn_EDTA_ (0.916), Pb (0.898), Pb_EDTA_ (0.879), S (0.628), P_OLSEN_ (−0.518)
F2	13.8	Ca (0.862), Tl (0.826), Mg (0.783), A/AC horizon thickness (−0.671)
F3	11.6	C_ORGANIC_ (0.87), N (0.789), Tl_EDTA_ (0.705)
F4	11.2	Silt (0.923), sand (−0.798)
F5	9.8	Clay (0.912), K (0.833), sand (−0.509)
F6	7.6	pH (−0.763), P (0.762)

### Relationship between habitat properties and vegetation

DFF and six factors representing soil properties were predictors of plant community parameters in multiple regression. All predictors were largely independent of each other: the factors were orthogonal and they did not correlate significantly with DFF. Table 4 shows the results of the multiple regression analysis.

**Table 4:** Effects of the distance from the forest and soil properties (represented by factors; see Table 3) on the plant species richness and composition (DCA 1, DCA 2), illustrated by standardized (Beta) coefficients of multiple regression

Dependent variable	N	Regression summary		Beta coefficients						
		*R* ^*2*^	*P*	DFF	F1	F2	F3	F4	F5	F6
Species richness	62	0.207	0.0040	0.15	−0.30*	0.24*	0.18	n.i.	0.20	−0.29*
DCA1	62	0.689	0.0000	−0.19*	−0.73***	−0.38***	0.08	0.14	−0.09	0.12
DCA2	61	0.281	0.0001	0.25*	n.i.	n.i.	−0.17	0.17	n.i.	−0.38**

*N* number of cases (outliers were removed), *R* ^*2*^ adjusted *R* ^*2*^, *P* significance level, *n.i.* indicates that the variable was not included in the model. Significant coefficients are marked with asterisks (* *P* < 0.05; ** *P* < 0.01; *** *P* < 0.001)

The properties of the habitat explained 21 % of the variation in species richness, and 69 and 28 % of the variation in species composition represented by DCA 1 and DCA 2 scores, respectively. Heavy metal contamination (represented by F1) was the most important determinant of plant community structure. It slightly reduced the number of species and dramatically altered the vegetation. Simple correlations between total Cd, Pb and Zn and selected plant species (Table 5) showed that increasing heavy metal stress caused the disappearance of metal-sensitive plants (typical of *CB*), such as *Brachypodium pinnatum*, and the emergence of metal-tolerant ones (typical of *CBF*), including *Carex caryophyllea* and *Festuca ovina*. The amount of carbonate minerals (represented by F2), which was high in shallow soils, had a positive influence on the species richness and affected the species composition (DCA 1) in a similar way as heavy metals. Soil acidity and the associated P concentration (represented by F6) had a negative effect on species richness, and also significantly determined the species composition (DCA 2). DFF had no effect on the species richness but significantly shaped the species composition (DCA 1 and DCA 2). According to Table 5, this variable was relevant to the few species: *Rubus caesius* (plant characteristic of ruderal and forest edge habitats) and *Lotus corniculatus* and *Carlina acaulis* (plants associated with grassland habitats).

**Table 5:** Spearman’s rank correlations between diagnostic and dominant species and selected habitat variables

Species	Community variant	Frequency	Spearman’s coefficients					
			DFF	A/AC horizon thickness	Ca	Cd	Pb	Zn
*Brachypodium pinnatum*	*CB*	22	0.19	0.45*	−0.23	−0.53*	−0.47*	−0.55*
*Helianthemum nummularium*	*CB*	19	0.13	0.26*	−0.19	0.09	0.20	0.04
*Coronilla varia*	*CB*	18	−0.10	0.13	−0.31*	−0.09	−0.02	−0.13
*Anthyllis vulneraria*	*CB*	17	0.18	−0.38*	0.36*	0.03	0.04	0.02
*Centaurea scabiosa*	*CB*	13	0.15	−0.02	0.05	−0.39*	−0.35*	−0.35*
*Knautia arvensis*	*CB*	13	0.02	0.09	−0.08	−0.14	−0.11	−0.19
*Polygala comosa*	*CB*	11	0.14	−0.06	0.19	−0.27*	−0.29*	−0.32*
*Carlina acaulis*	*CB*	9	0.26*	0.07	0.16	−0.25*	−0.32*	−0.24
*Carex caryophyllea*	*CBF*	41	0.09	−0.04	0.03	0.42*	0.40*	0.40*
*Thymus pulegioides*	*CBF*	35	0.12	−0.23	0.19	0.36*	0.29*	0.36*
*Scabiosa ochroleuca*	*CBF*	34	0.17	−0.02	0.17	0.11	0.05	0.07
*Festuca ovina*	*CBF*	33	0.13	−0.45*	0.21	0.58*	0.45*	0.60*
*Dianthus carthusianorum*	*CBF*	32	0.08	−0.20	−0.03	0.45*	0.31*	0.48*
*Potentilla arenaria*	*CBF*	30	0.10	−0.32*	0.34*	0.25*	0.14	0.25*
*Festuca rubra*	*CBR*	22	−0.06	0.02	−0.16	−0.20	−0.08	−0.19
*Rubus caesius*	*CBR*	18	−0.32*	0.10	−0.24	−0.42*	−0.27*	−0.39*
*Galium album*	–	46	−0.08	−0.03	−0.08	0.35*	0.33*	0.38*
*Euphorbia cyparissias*	–	45	0.06	−0.12	0.01	−0.09	−0.09	−0.07
*Lotus corniculatus*	–	33	0.45*	−0.06	0.22	0.00	−0.09	0.04
*Pimpinella saxifraga*	–	31	−0.02	−0.24*	0.13	0.22	0.17	0.17
*Leontodon hispidus*	–	28	0.08	−0.32*	0.31*	0.20	0.05	0.24
*Medicago falcata*	–	26	0.03	0.13	−0.15	−0.34*	−0.36*	−0.29*
*Achillea collina*	–	23	0.04	0.02	−0.12	−0.21	−0.13	−0.21
*Plantago lanceolata*	–	23	0.05	−0.15	0.07	0.11	0.07	0.13
*Silene vulgaris*	–	23	−0.02	−0.38*	0.22	0.59*	0.46*	0.59*
*Carex hirta*	–	20	−0.14	−0.11	0.09	0.49*	0.38*	0.49*
*Vicia cracca*	–	20	−0.07	0.24	−0.03	0.15	0.14	0.10

The list of species includes those diagnostic of the typical variant of *Carlino acaulis*-*Brometum erecti* (*CB*), the *Festuca ovina* variant (*CBF*) and the *Rubus caesius* variant (*CBR*), according to Chytrý (2007), and other frequent plants (recorded in at least 30 % of sites). Significant coefficients (*P* < 0.05) are asterisked

*DFF* distance from the forest

## Discussion

This study showed that the amount of heavy metals (Cd, Pb and Zn) in the soil of old mining heaps strongly determined the species composition of plant communities growing there. As expected, the number of metal-sensitive species decreased, while the number of metal-tolerant increased with increasing heavy metal contamination. As a result, the typical dry calcareous grassland (dominated by *Brachypodium pinnatum*; *CB*) was replaced by its heavy metal variant (dominated by *Festuca ovina*; *CBF*). The vegetation encroaching on heavily contaminated soil (*CBF*) was mainly composed of common grassland species with a wide ecological amplitude, but of low competitiveness, for example, *Carex caryophyllea*, *Festuca ovina*, *Silene vulgaris*, *Thymus pulegioides*, *Dianthus carthusianorum* or *Potentilla arenaria*. According to the literature (Ernst 2006), such species can produce ecotypes able to colonize metalliferous substrates. Some of them (e.g. *Festuca ovina*, *Silene vulgaris*) are regarded as facultative metallophytes, characteristic of Central European heavy metal grasslands (Ernst 1974; Punz and Mucina 1997; Brown 2001; Szarek-Łukaszewska and Grodzińska 2011).

Analysis of plant community in terms of life forms and life history strategies can be helpful in understanding the nature of the selection pressure acting on plant populations (Grime 2001). For example, a significant share of stress tolerators, chamaephytes and plants dispersed by wind may be a response to harsh site conditions, which happens in young, disturbed habitats (Grime 2001; Řehounková and Prach 2010). In contrast, the prevalence of competitors, phanerophytes and plants dispersed by animals indicates less extreme situations typical of late-successional stages (Olsson 1987; Grime 2001; Novák and Prach 2003; Řehounková and Prach 2010). Following these guidelines, the *CBF* habitats can be considered more hostile in comparison with others, especially the *CBR* habitats. However, these differences are small, which probably reflects the close relationship between the grasslands surveyed.

Multiple regression showed that the total Ca and Mg could be relevant predictors of plant species composition. These variables negatively correlated with the thickness of A/AC horizon, as indicated by factor analysis, thus it reflected to some extent the development of the soil (the shallower the soil, the more Ca- and Mg-rich mining waste minerals in soil samples). The importance of this factor in shaping the structure of grassland communities becomes apparent in the analysis of stratified data (excluding the sites with high soil concentrations of Cd, Pb and Zn). The thickness of A/AC horizon, under low and moderate heavy metal contamination, was the primary determinant of the plant species composition: the *CBF* grassland developed on shallow, often skeletal soil, while the *CB* grassland on well-developed soil. This means that the *CBF* grassland, under certain environmental conditions (lower heavy metal stress), can be succeeded by the *CB* grassland along with the ongoing process of soil formation.

The distance from the forest (DFF) was another parameter that caused substantial changes in the structure of grassland communities studied. This is in line with many authors (Butaye et al. 2002; Novák and Konvička 2006; Prach and Řehounková 2006; Pen-Mouratov et al. 2014) who found that the pattern of primary succession in man-made island habitats was dependent on the distance from other habitats; these authors concluded that new sites were effectively colonized by vegetation growing no further than a few dozen meters away. In this study, the proximity of the forest triggered a transition from the typical *CB* community into its *CBR* variant. This was probably due to increased seed rain of forest species (McDonnell and Stiles 1983; Myster and Pickett 1993), including competitive woody plants. Also, the deposition of tree litter may play an important role in structuring the plant community (note that the mean DFF was 28 m, which means that many *CBR* sites were adjacent to forest), since it can inhibit the germination of some plants and increase the availability of some nutrients (Loydi et al. 2013). The latter effect was barely visible in terms of soil properties—the *CBR* soil did not differ from other soils in the total N and P concentrations, organic C concentration or thickness of the O horizon; it only had elevated levels of available P. However, the emergence of nutrient-demanding and synanthropic plants (e.g. *Rubus caesius* and *Solidago canadensis*) suggests that the supply of nutrients is to be taken into account in explaining the species composition of the *CBR* community.

The number of plant species is a parameter responsive to environmental changes (Tilman 1988; Grime 2001). However, in this study, it did not correlate (or correlated poorly) with the majority of the habitat characteristics (e.g. pH or clay content). The reason for this was likely limited variability of heap habitats. This did not apply to the concentrations of heavy metals, which varied largely among sites. In this case, lack of significant differences in species richness between *CB*, *CBF* and *CBR* grasslands and the relatively weak relationship between heavy metals and species richness can be explained by the interaction of metal toxicity and species composition. High heavy metal contamination eliminated some species, but at the same time, it promoted the growth of others due to reduction of interspecific competition; low heavy metal contamination was not harmful for plants, hence it allowed metal-sensitive competitors (e.g. *Brachypodium pinnatum*) to survive and dominate the community. A similar mechanism has been described for plant communities affected by natural disturbances such as grazing or soil perturbation by animals (Bobbink 1991; Barbaro et al. 2004; Questad and Foster 2007; Stevens et al. 2010; Kurek et al. 2014).

## Conclusions

Structure of the grassland community that colonizes sites of historical Zn–Pb mining (heaps) is determined primarily by the amount of heavy metals in the soil. With increasing heavy metal stress, metal-sensitive plants withdraw. This loss of species is partly compensated by the emergence of metal-tolerant plants. As a result, typical dry grassland changes into heavy metal grassland, while the species richness remains relatively constant.

Metal-tolerant vegetation may develop under low- and high-contamination conditions. In the former case, it occupies heaps with shallow, skeletal soil. In contrast, metal-sensitive vegetation grows on heaps with well-developed soil. These observations suggest that heavy metal grasslands colonizing substrates with relatively low metal toxicity are ephemeral communities, and tend to succeed over time. The direction of this succession is affected by the surrounding vegetation. Under high-contamination conditions, this process seems to be greatly slowed down.
